# Metal-Size Influence in Iso-Selective Lactide Polymerization**

**DOI:** 10.1002/anie.201403643

**Published:** 2014-07-07

**Authors:** Clare Bakewell, Andrew J P White, Nicholas J Long, Charlotte K Williams

**Affiliations:** Department of Chemistry, Imperial College LondonSouth Kensington (UK)

**Keywords:** iso-selectivity, lactide, lanthanum, lutetium, ring-opening polymerization

## Abstract

Iso-selective initiators for the ring-opening polymerization (ROP) of *rac*-lactide are rare outside of Group 13. We describe the first examples of highly iso-selective lutetium initiators. The phosphasalen lutetium ethoxide complex shows excellent iso-selectivity, with a *P*_i_ value of 0.81–0.84 at 298 K, excellent rates, and high degrees of polymerization control. Conversely, the corresponding La derivative exhibits moderate heteroselectivity (*P*_s_=0.74, 298 K). Thus, the choice of metal center is shown to be crucial in determining the level and mode of stereocontrol. The relative order of rates for the series of complexes is inversely related to metallic covalent radius: that is, La>Y>Lu.

Polylactide (PLA), a degradable polymer obtained from renewable resources, is one of the leading commercial alternatives to petrochemical plastics.[1] PLA is produced by the metal-catalyzed ring-opening polymerization (ROP) of lactide (LA).[2] The central challenge in this field of catalysis is to combine high rates with excellent stereocontrol, ideally without the need for expensive chiral auxiliaries or ligands.[3] Iso-selectivity using *rac*-LA is especially important and useful because the product, stereoblock/complex PLA, has superior properties.[4] For example, it is a crystalline polymer with a higher melting temperature (*T*_m_) and better mechanical properties than isotactic poly-L-lactide (PLLA). In some cases, *T*_m_ is elevated by as much as 50 °C, greatly improving thermal stability and enabling PLA to compete as an engineering polymer.[5] However, *rac*-LA iso-selective catalysts remain scarce, with the majority being chiral aluminum salen complexes or derivatives.[3b, 4a], [6] Although these compounds show impressive degrees of stereocontrol, they are often extremely slow and require unacceptably high catalyst loadings, typically taking hours or days to reach completion even at 1 mol % catalyst loading. There are only a handful of other, non-aluminum based, iso-selective catalysts,[7] the structures of three of the most selective of these are shown in Figure 1.[8] In 2008, Arnold and co-workers reported a homochiral yttrium complex that showed good iso-selectivity (**C**, *P*_i_=0.75, 298 K).[8a,b] Subsequently, we reported yttrium phosphasalen complexes (Figure 1, structures **A** and **B**), which combined very high rates with promising iso-selectivity (*P*_i_=0.74, 298 K).[8c]

**Figure 1:**
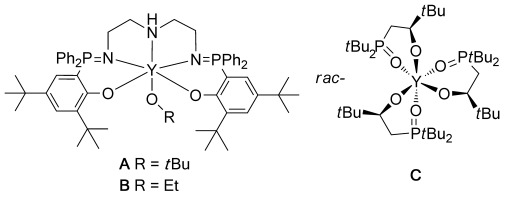
The structure of iso-selective yttrium phosphasalen complexes A and B[8c] and a homochiral lanthanide complex C, reported by Arnold et al.[8a]

Herein, we report the performance of lutetium and lanthanum phosphasalen complexes. For such Group 3 lanthanide complexes, the coordination geometries are predominantly influenced by steric factors. In this regard, it is relevant that lutetium has a slightly smaller covalent radius than yttrium (1.87 Å versus 1.90 Å), whilst lanthanum is significantly larger (2.07 Å).[9]

The use of different lanthanide centers enables an investigation of the influence of atomic size and coordination environment on polymer tacticity. Both metals have precedence in lactide ROP catalysis,[10] although lutetium is rarely investigated.[10b,e,f,h] Okuda and co-workers reported heteroselective (syndiotactic) dithiaalkanediyl-bridged bis(phenolato) yttrium and lutetium catalysts, but found that the stereocontrol decreased from Y to Lu.[10b,f] To our knowledge, there is no precedent for any iso-selective lutetium initiators.

The initiators were prepared in good overall yields, from the phosphasalen ligand, which was synthesized using an established procedure (Scheme 1).[8c] First, the ligand was deprotonated using potassium bis(trimethylsilyl)amide (5 equivalents), leading to quantitative conversion into the salt, as observed by the upfield shift (*δ*_P_=23 ppm) of the signal for the phosphorus center in the ^31^P{^1^H} NMR spectrum. The lutetium phosphasalen chloride complex was formed, but not isolated, by reaction with LuCl_3_. One signal was observed for this species at *δ*=35 ppm in the ^31^P{^1^H} NMR spectrum. Addition of the relevant potassium alkoxide (ethoxide or *tert*-butoxide) led to the formation of the lutetium phosphasalen alkoxide complexes (**1** and **2**). A slight upfield shift was again observed for resonances in the ^31^P{^1^H} NMR spectra of both complexes (*δ*_P_=34 ppm). Compounds **1** and **2** were isolated in good yields after recrystallization (55 % and 75 %, respectively).

**Scheme 1:**
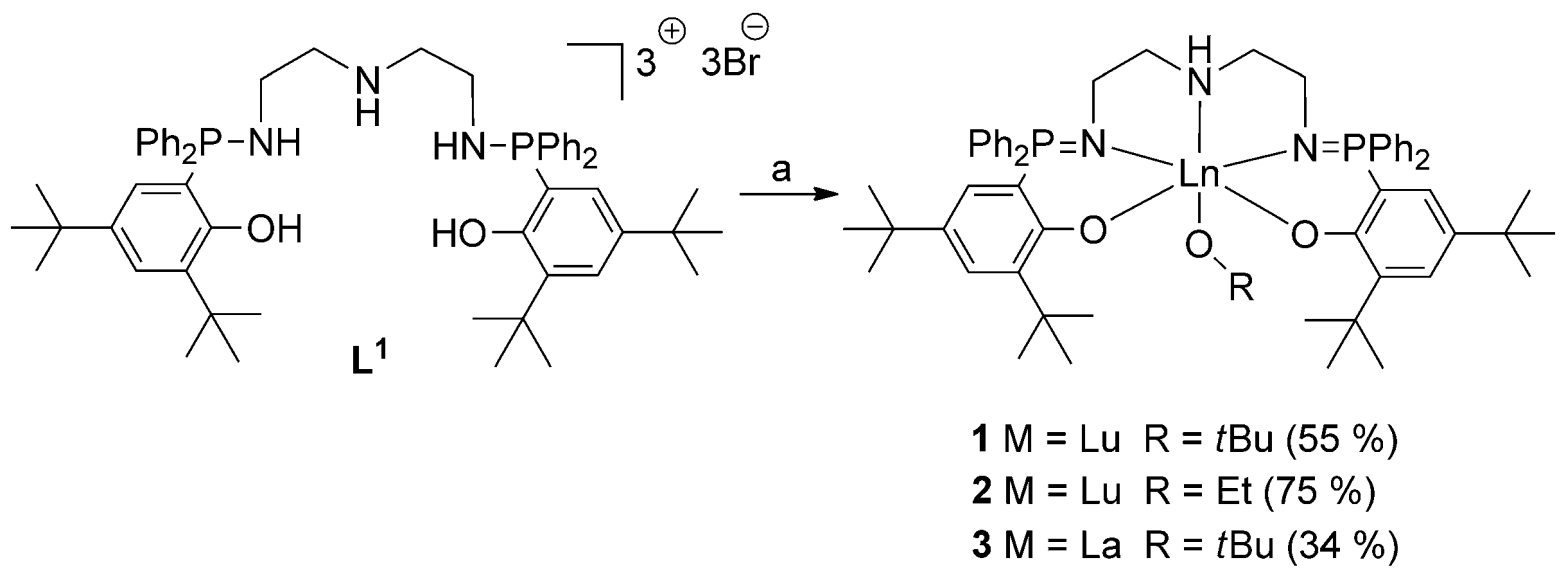
Synthesis and structure of initiators 1–3. a) Reaction conditions: 1) KN(SiMe_3_)_2_ (5 equiv), THF, 2 h, 298 K; 2) LuCl_3_ or LaCl_3_, THF, 4 h, 298 K; 3) KOR, THF, 4 h, 298 K.

The complexes were fully characterized by NMR spectroscopy and elemental analysis. At room temperature, the ^1^H NMR spectra of both **1** and **2** show broad resonance signals, indicative of fluxional processes, however, at 360 K coalescence occurs and clearly distinguishable signals are observed (see Figure S9 in the Supporting Information). At 298 K, the ^31^P{^1^H} NMR spectrum of compound **1** shows two signals in a 1:7 ratio, but on heating to 360 K a single sharp signal is observed at *δ*=34 ppm. The phosphasalen lanthanum *tert*-butyl alkoxide complex (**3**) was formed in an analogous fashion, albeit in a slightly lower yield (34 %). The intermediate chloride complex showed a single signal at *δ*=33 ppm in the ^31^P{^1^H} NMR spectrum. Addition of potassium *tert*-butoxide led to the formation of **3**, which exhibited a single peak at lower chemical shift (*δ*_P_=30 ppm). Crystals of all three new complexes suitable for single-crystal X-ray diffraction were isolated from solutions of the complexes in mixtures of cyclohexane and hexane (note that complex **3** crystallized with two independent molecules, **3** **A** and **3** **B**).

The structures of the complexes (Figure 2, Figures S1–S4) all show a severely distorted octahedral geometry at the metal center with *trans* angles in the ranges 144.52(14)–158.87(12)°, 141.9(3)–159.3(3)°, 127.11(16)–149.44(13)°, and 123.71(18)–146.96(13)° for **1**, **2**, **3** **A**, and **3** **B**, respectively. The yttrium analogue **A** ^[8c]^ has a similar geometry to those observed for **1** and **2**. In each case, the pentacoordinate ligand occupies one hemisphere, leaving the alkoxide ligand isolated in the other hemisphere (Figure 2). The geometries of **1** and **2** are very similar, as would be expected with the only difference between the two complexes being a change in the alkoxide co-ligand. The lanthanum structure **3**, however, shows marked differences. In addition to the expected elongation of all of the M=X bonds (Table S1) and the even greater distortion from ideal octahedral coordination angles at the metal center (see above), the O1⋅⋅⋅O21 phenoxide⋅⋅⋅phenoxide separation is markedly increased (3.074(4), 3.107(9), 3.154, 3.603(5), and 3.354(5) Å, measured for **1**, **2**, **A** (yttrium analogue),[8c] **3** **A**, and **3** **B**, respectively). Similarly, the “hole” in which the alkoxide ligand sits is larger in **3** **A** and **3** **B** than in either **1**, **2**, or yttrium analogue **A**.[8c] The closest approaches between the methyl group of the alkoxide and a carbon atom of the proximal phenyl ring are approximately 3.74, 3.83, 3.81, 4.67, and 4.13 Å for complexes **1**, **2**, **A**,[8c] **3** **A**, and **3** **B**, respectively. All four structures adopt asymmetric conformations that place one phenyl ring of one of the two PPh_2_ units—specifically a phenyl ring attached to P15—much closer to the alkoxide than the other three phenyl rings. There are noticeable differences in geometry between **3** **A** and **3** **B**, which are chemically identical, suggesting a distinct degree of flexibility in both the ligand and the coordination environment in **3**. The most visually obvious difference is the orientation of the C48-containing phenyl ring bound to the P15 atom (the phosphorus closest to the alkoxide); in **3** **A** this ring is oriented approximately orthogonally to the N14=P15 bond, whereas in **3** **B** it is almost parallel to this bond (see Figures S3, S4).

**Figure 2:**
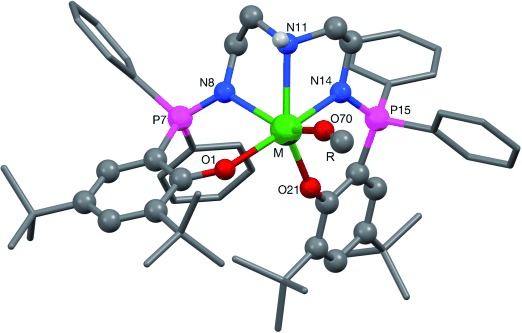
Schematic representation of the single-crystal X-ray structures of complex 1 (M=Lu, R=*t*Bu), 2 (M=Lu, R=Et) and 3 (molecules A and B, M=La, R=*t*Bu).

Compounds **1**–**3** were tested as initiators for the ROP of *rac*-LA. Initially the experiments were conducted under standard conditions, using a 1 m solution of LA in THF at 298 K and with a 2 mm concentration of initiator. The progress of the polymerization was monitored by taking regular aliquots. All the new compounds (**1**–**3**) showed good to very good activities (Table 1). Based on the time required to achieve complete conversion of *rac*-LA into PLA, the order of activity decreased: La>Y>Lu. To quantify these observations, the polymerization kinetics were monitored (Figures S11, S12).

**Table 1:** Polymerization data obtained using initiators 1–3 in THF, 298 K, [LA]=1 m.

I	[I]:[*i*PrOH]:[LA]	*t* [h]	Conversion [%][d]	*M*_n,exp_ [g mol^−1^][e]	*M*_n,calcd_ [g mol^−1^]	PDI[e]	*P*_i_[f]
**1**	1:500[g]	8	81	101 700	58 300	1.06	0.80
**1**	1:1:500	9	84	38 900	60 500	1.07	0.75
**1**[ac]	1:0.5:500	72	84	69 600	60 500	1.02	0.84
**1**[bc]	1:0.5:200	48	90	36 000	26 000	1.02	0.83
**2**	1:500[g]	8.25	86	53 400	61 900	1.02	0.82
**2**	1:350[g]	5.5	86	38 300	43 300	1.09	0.82
**2**	1:250[g]	3.5	86	34 900	31 000	1.02	0.84
**2**	1:200[g]	2.75	89	27 800	25 600	1.05	0.81
**2**[ac]	1:500[g]	72	75	46 300	54 000	1.01	0.89
**2**[bc]	1:200[g]	48	81	22 800	23 300	1.02	0.89
**3**[a]	1:1:500	20 s	98	57 300	70 600	1.05	0.28
**3**	1:2:1000	20 s	93	58 000	67 000	1.03	0.28

[a] 0.75 m [LA].

[b] 0.5 m [LA].

[c] 257 K.

[d] Determined by integration of the methine region of the ^1^H NMR spectrum (LA, *δ*=4.98–5.08 ppm; PLA, *δ*=5.09–5.24 ppm).

[e] Determined by GPC (gel permeation chromatography) in THF versus polystyrene standards (*M*_n_ values are corrected with a 0.58 factor).[11]

[f] Determined by analysis of the homonuclear decoupled NMR spectrum according to the method first described by Coudane et al.[12]

[g] No *i*PrOH added. I=initiator.

In all cases, a first-order dependence of the conversion rate on lactide concentration was observed, as evidenced by the linear fit of data to plots of ln([LA]_t_/[LA]_0_) versus time, from which the gradient corresponds to the *k*_obs_ value. For **1** and **2**, the *k*_obs_ values are comparable at 6.3×10^−5^ s^−1^ and 7.5×10^−5^ s^−1^, respectively (Figure S11). This similarity is in line with expectations as the two compounds differ only in the nature of the alkoxide which is the initiating group. The two lutetium complexes **1** and **2** are approximately an order of magnitude slower than yttrium complexes **A** and **B** (*k*_obs_=6.9×10^−4^, 7.9×10^−4^ s^−1^). This finding is consistent with other examples of lutetium complexes.[10b,f,h,j], [13] In contrast, lanthanum complex **3** was an extremely fast initiator, enabling almost complete conversion of 1000 equivalents of LA [versus initiator, in the presence of isopropyl alcohol (2 equivalents)] in less than 20 seconds (Table 1). Such rates are beyond the limit of polymerization monitoring using aliquots and so no further kinetics studies were undertaken. Instead, the rate law for polymerization using **2** was investigated; *k*_obs_ values were determined over a range of different initiator concentrations, ([**2**]=2–5 mm, at 298 K maintaining [LA]=1 m). In each case, the reactions were first-order in lactide concentration. The reactions also showed a first-order dependence on the concentration of initiator **2**, as shown by the linear fit to the plot of *k*_obs_ value versus the concentration of **2** (Figure 3). Thus, the overall rate law is second-order and the propagation rate constant, *k*_p_, is 5.08×10^−5^ m^−1^ s^−1^. As the rate depends on both initiator and lactide concentrations, the rate-limiting step could correspond to LA coordination or insertion, or a composite of both. Given that the order of rate (La>Y>Lu) is inversely related to the metal covalent radii and the expected Lewis acidities, it is hypothesized that the rate-limiting process is lactide insertion into the metal–alkoxide bond. Accordingly, the selection of the metal center controls the competing requirements for efficient polymerization, that is, the need for sufficient Lewis acidity to ensure rapid coordination, balanced with an alkoxide bond which is labile to substitution. The order of rates correlates with the metal–alkoxide bond lengths, as determined by single-crystal XRD experiments. The lanthanum–alkoxide bond is significantly longer than the lutetium–alkoxide and yttrium–alkoxide bonds (M–O21: 2.196(4), 2.060(3), 2.069(6) Å in **3** **A** (La), **1** (Lu), and **A** (Y), respectively).

**Figure 3:**
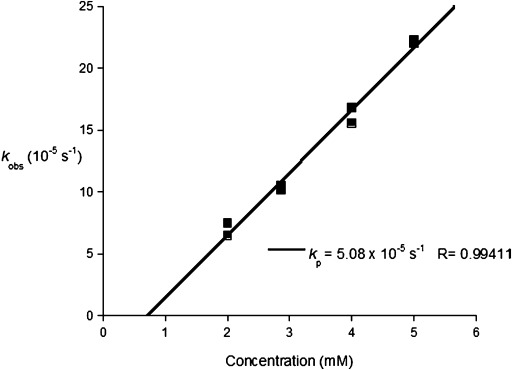
Plot of *k*_obs_ value versus [2]. Polymerization conditions: [LA]_0_=1 m, [2]=2–5 mm,THF, 298 K. Average errors: 1–8 %.

Despite the high overall rates, the polymerizations remained very well controlled. In general, there was a close agreement between the theoretical and observed molecular weights, and the PLA had a narrow polydispersity index (PDI<1.10 in all cases, Table 1). Complexes **1** and **3** contain *tert*-butyl alkoxide as the co-ligand. This hindered alkoxide has been shown to undergo relatively slower initiation leading to *M*_n_ values exceeding those predicted.[8c] To overcome this limitation, isopropyl alcohol (0.5–1 equivalents) was added, which undergoes rapid and reversible exchange reactions leading to faster initiation and, in the case of **1**, a very good match between the calculated and the experimental *M_n_* values. In contrast, complex **2**, containing an ethoxide co-ligand, enables very well-controlled polymerization without the need for any exogeneous alcohol.

Importantly, in addition to high rates and good polymerization control, the lutetium complexes **1** and **2** both polymerize *rac*-LA with a high iso-selective bias, as assessed by the *P*_i_ values which were determined by integration (normalized) of the homonuclear decoupled NMR spectra and comparison with the values predicted by Bernouillan statistics.[12] Compound **1** showed a significantly improved iso-selectivity compared to compound **A** (yttrium analogue) at 298 K (*P*_i_=0.80 versus *P*_i_=0.75). However, when one equivalent of isopropyl alcohol was added, the degree of iso-selectivity dropped (*P*_i_=0.75 for **1**, versus *P*_i_=0.73 for compound **A** with *i*PrOH), presumably because of some scrambling of stereochemistry during chain transfer. Compound **2**, which does not require any alcohol, was even more iso-selective at room temperature (*P*_i_=0.82±0.02) and represents, to our knowledge, the highest iso-selectivity catalyst reported, excluding the aluminium salen complexes. As mentioned, although *P*_i_ values exceeding 0.95 are known for these chiral aluminum salen ligands, their activities are very low and the high loadings (more than 1 mol %) limit their applicability.

We next considered how to control the iso-selectivity. The *P*_i_ value remained high over a range of different catalyst loadings and therefore enabled the production of rather high *M*_n_ stereoblock PLA (Table 1). Stereocontrol is a kinetic phenomenon, therefore reducing the reaction temperature enables an increase in iso-selectivity, leading to a maximum *P*_i_ value of 0.89 at 257 K. Analysis of the defect tetrad signals in the homonuclear decoupled NMR spectrum indicate that iso-selectivity arises from a chain-end control mechanism (approximately a 1:1:1 ratio of sii:iis:isi; Figure S14). Differential scanning calorimetry (DSC) indicated the formation of semi-crystalline PLA with a *T_m_* of 178 °C (Figure S16). The increased degree of iso-selectivity for complex **1** and **2** (versus yttrium analogues **A** and **B**) is tentatively proposed to be as a result of the smaller lutetium metal center enforcing a more sterically encumbered bonding geometry by the phosphasalen ligand.

Thus, we were surprised to discover that the lanthanum initiator, **3**, exhibited moderate heteroselectivity (*P*_s_=0.72). This is unexpected as a gradual decrease in stereocontrol on decreasing steric shielding is much more usual.[10c,d] We have previously observed that for a series of yttrium phosphasalen complexes, the stereocontrol switches from heterotactic (for tetracoordinate ligands) to isotactic (for pentacoordinate ligands).[8c] In contrast, the same pentacoordinate ligand bound herein to a series of lanthanide centers forms compounds with very closely related coordination geometries (see above), which leads to completely opposite stereoselectivities depending on the metal size. Insight into the propensity for an initiator to exhibit iso-selectivity or heteroselectivity can be gained by analysis of the NMR spectra. High-temperature ^1^H NMR experiments of the lutetium compounds show that signal coalescence is reached by 360 K (Figure S5, S6). Under the same conditions, resonance signals attributable to the lanthanum complex are still significantly broadened, indicating a more fluxional structure (Figure S7). 2D NMR spectroscopic experiments were even more informative. By using rotating-frame nuclear Overhauser effect spectroscopy (ROESY), it is possible to compare the fluxionalities of the complexes by monitoring proton-exchange events. It is important to note that all complexes showed proton exchange in the iminophosphorane bridges, in line with the broadened resonance signals in the ^1^H NMR spectra observed at 298 K. Lutetium complexes **1** and **2** showed limited further exchange, as did the yttrium analogues **A** and **B**.[8c] However, the lanthanum analogue **3** also showed significant exchange of the phenyl ring protons associated with the PPh_2_ groups (Figures S17, S18). These exchange events correspond to the protons experiencing the same magnetic environment on the NMR timescale, in this case they are indicative of rotations of the phenyl groups. Such rotations are consistent with **3** exhibiting a significantly more fluxional structure than **1** and **2**. Interestingly, the rotations of the phenyl groups coordinated to the phosphorus are also manifested in the solid-state structures of the two independent molecules of compound **3**: **3** **A** and **3** **B** (Figures S3, S4). Furthermore, previous studies using different yttrium derivatives, revealed that heteroselective catalysts exhibited free rotation of the phenyl groups whereas the iso-selective yttrium catalysts **A** and **B** showed no such fluxionality.[8c] Thus, these NMR studies add further weight to the hypothesis that iso-selectivity results from sterically congested metal complexes with limited fluxionality of the phosphorus substituents. In contrast, heteroselectivity is observed when using the larger metal center (La) which shows a more open coordination geometry and fluxionality of the phosphorus substituents.

In conclusion, we have reported highly iso-selective lutetium initiators. The phosphasalen lutetium ethoxide complex shows a *P*_i_ value of 0.82±0.02 at 298 K, moving to 0.89 at 257 K. In contrast, the analogous lanthanum initiator was heteroselective (*P*_s_=0.72, 298 K). All initiators show excellent rates and high degrees of polymerization control. Both the relative order of rates and the mode and degree of stereocontrol for the series of complexes have been shown to be related to the metallic covalent radius. A larger metal center results in a higher observed rate (La>Y>Lu). The smaller metal center of lutetium promotes high iso-selectivity (higher than the previously reported yttrium), whilst the more open coordination geometry of lanthanum leads to moderate heteroselectivity. The type of stereocontrol appears to be associated with the level of rigidity imposed on the ligand. Thus, from a single ligand, two different modes of stereocontrol are possible: such switching is very unusual and warrants further investigation as an attractive route to control polymer properties.
