# Supplementary material for: Metal-Size Influence in Iso-Selective Lactide Polymerization
Source: Angew Chem Int Ed Engl. 2014 Jul 7;53(35):9226–30. doi: 10.1002/anie.201403643 (PMC4499264; doi:10.1002/anie.201403643)
Supplement: Supplementary file 1 [file anie0053-9226-sd1.pdf]

Angewandte Chemie
Eine Zeitschrift der Gesellschaft Deutscher Chemiker

Supporting Information
© Wiley-VCH 2014
69451 Weinheim, Germany

Metal-Size Influence in Iso-Selective Lactide Polymerization**

Clare Bakewell, Andrew J. P. White, Nicholas J. Long, and Charlotte K. Williams**

anie_201403643_sm_miscellaneous_information.pdf

Supporting Information

Pages 2-4: Experimental Section

Pages 5-9: X-ray Crystallographic data (Figs. S1-S4, Table S1)

Pages 10-12: NMR spectra of compounds **1-3** (Figs. S5-S10)

Pages 13-14: Polymerization data using initiators **1-3** (Figs. S11-S13)

Page 15: Homonuclear decoupled ^{1}H{^{1}H} NMR spectra (Figs. S14-S15)

Page 16: DSC data (Fig. S16)

Page 17: ROESY NMR spectra (Figs. S17-S18)

Pages 18-19: Polymerization data tables (Tables S2-S4)

Page 20: References

Experimental Section

Materials and Methods

All reactions were conducted under an atmosphere of dry nitrogen, or argon, using standard Schlenk line and glovebox techniques. Solvents and reagents were obtained from commercial sources. Tetrahydrofuran, toluene and hexane were distilled from sodium/benzophenone, under dry nitrogen. Cyclohexane was dried over 3Å molecular sieves. Dichloromethane was distilled from CaH_2, under dry nitrogen. *Rac*-lactide was recrystallized from anhydrous toluene and sublimed under vacuum three times prior to use. Lutetium (III) chloride and lanthanum (III) chloride were obtained from Strem Chemicals. The phosphasalen pro-ligand was prepared according to previously reported literature procedures.[1]

Nuclear magnetic resonance (NMR) spectra were recorded on a Bruker Av300 instrument. Solvent peaks were used as internal references for ^{1}H and ^{13}C chemical shifts (ppm). ^{31}P peaks were referenced to external 85% H_3PO_4. When needed, higher resolution ^{31}P{^{1}H} NMR and ^{1}H{^{1}H} NMR (homo-decoupled spectroscopy) experiments were performed on a Bruker Av500 spectrometer, equipped with a z-gradient bbo/5 mm tuneable probe and a BSMS GAB 10 A gradient amplifier providing a maximum gradient output of 5.35 G/cmA. ^{1}H NMR spectra for all lactide polymerizations were performed on a Bruker Av4000 or Av500 instrument. The following abbreviations are used: br, broad; s, singlet; d, doublet; dd, doublet of doublets; t, triplet; m, multiplet. Rotating frame nuclear Overhauser effect spectroscopy (ROESY) were conducted by Mr Peter Haycock on a Bruker 500MHz AVANCE III HD spectrometer running TopSpin 3.2 and equipped with a z-gradient bbo/5mm tuneable SmartProbe™ and a GRASP II™ gradient spectroscopy accessory providing a maximum gradient output of 53.5G/cm (5.35G/cmA). The Bruker pulse program roesyetgp was employed.[2]

Elemental analyses were determined by Mr. Stephen Boyer at London Metropolitan University. PLA number averaged molecular weight, M_n, and polydispersity index (M_w/M_n; PDI) were determined using gel permeation chromatography (GPC). Two Polymer laboratories Mixed D columns were used in series, with THF as the eluent, at a flow rate of 1 mL min^{-1}, on a Polymer laboratories PL GPC-50 instrument at 40 °C. Polymer molecular number (M_n) was determined by comparison against polystyrene standards, using correction factor of 0.58 as reported by Penczek and Duda.[3] PLA stereochemistry was determined by comparison of the normalized integrals for all the tetrad signals in the homonuclear proton decoupled NMR spectrum. The tetrad signals' integrals were compared against the values predicted by Bernoullian statistics,[4] so as to enable determination of the probability of an iso-tactic diad (P_i) to be determined for each tetrad signal. The average P_i value from all 5 signals is reported. The peaks were integrated using peak deconvolution and the values normalised, deconvolution was achieved using Mestrenova software.

Compound 1

Potassium *bis*(trimethylsilyl)amide (533 mg, 2.60 mmol) was added into a slurry of the phosphasalen pro-ligand (0.60 g, 0.50 mmol) in THF (15 mL). After 2 h, a cloudy suspension formed. The completion of the deprotonation reaction was verified by ^{31}P{^{1}H} NMR spectroscopy. The insoluble potassium salt was removed by centrifugation and $LuCl_3$ (150 mg, 0.50 mmol) was added. After 4 h of stirring at 298 K, potassium *tert*-butoxide (60 mg, 0.50 mmol) was added into the mixture, giving a cloudy suspension. Stirring was continued for 4 h, after which time the solid was removed by centrifugation. The solvent was evaporated *in vacuo* and the residue was crystallised from cyclohexane (4 mL), giving compound **1** as colourless crystals (330 mg, 0.30 mmol, 55 %).

^{1}H NMR (400 MHz, toluene-d_8, 360 K) δ (ppm): 7.59-7.70 (bm, 8H, C*H*(PPh$_2$)), 7.50 (d, 2H, C$_b$*H*, $^4J_{H,H}$=2.6 Hz), 7.09-7.16 (bm, 8H, C*H*(PPh$_2$)), 7.00-7.05 (bm, 4H, C*H*(PPh$_2$)), 6.75 (dd, 2H, C$_d$*H*, $^4J_{H,H}$=2.6 Hz, $^3J_{P,H}$=16.0 Hz), 3.19 (m, 4H, P=N-C*H*$_2$-CH$_2$), 2.99 (m, 4H, P=N-CH$_2$-C*H*$_2$), 2.29 (bm, 1H, N*H*), 1.44 (s, 18H, *t*Bu), 1.26 (s, 9H, O*t*Bu), 1.19 (s, 18H, *t*Bu); ^{31}P{^{1}H} NMR (161.9 MHz, toluene-d_8, 360 K) δ (ppm): 34.1 (s, *P*); ^{13}C{^{1}H} NMR (100 MHz, benzene-d_6, 298 K) δ (ppm): 169.9 (d, $^2J_{P,C}$=3.6 Hz, *C*^{IV}-O), 140.7 (d, $^3J_{P,C}$=8.0 Hz, *C*$_{c,a}^{IV}$), 134.1 (d, $^3J_{P,C}$=15.0 Hz, *C*$_{c,a}^{IV}$), 133.6 (d, $^{2/3}J_{P,C}$=9.9 Hz, *m*-or *o*-*C*H(PPh$_2$)) 133.4 (d, $^{2/3}J_{P,C}$=9.0 Hz, *m*-or *o*-*C*H(PPh$_2$)), 132.3 (d, $^1J_{P,C}$=75.0 Hz, *C*^{IV}-P), 131.4 (s, *p*-*C*H(PPh$_2$)), 131.2 (s, *p*-*C*H(PPh$_2$)), 128.2 (d, $^{2/3}J_{P,C}$=12.0 Hz, *m*-or *o*-*C*H(PPh$_2$)), 128.1 (s, *C$_b$*H), 128.0 (d, $^{2/3}J_{P,C}$=12.0 Hz, *m*-or *o*-*C*H(PPh$_2$)), 127.2 (d, $^1J_{P,C}$=13.0 Hz, *C$_d$*H), 111.0 (d, $^1J_{P,C}$=116.0 Hz, *C*^{IV}-P), 70.5 (s, O-*C*^{IV}(CH$_3$)$_3$), 53.5 (d, $^2J_{P,C}$=16.0 Hz, P^{IV}-N-*C*H$_2$), 48.3 (d, $^3J_{P,C}$=4.5

Hz, P^{IV}-N-CH_2-CH_2), 35.9 (s, $C^{IV}(CH_3)_3$), 35.5 (s, O-$C^{IV}(CH_3)_3$), 34.2 (s, $C^{IV}(CH_3)_3$), 31.9 (s, $C^{IV}(CH_3)_3$), 30.3 (s, $C^{IV}(CH_3)_3$); Anal. Calc. ($C_{60}H_{78}N_3O_4P_2Lu$): C, 63.99; H, 6.98; N, 3.73. Found: C, 63.76; H, 7.02; N, 3.84.

Compound 2

Potassium *bis*(trimethylsilyl)amide (266 mg, 1.30 mmol) was added into a slurry of the phosphasalen pro-ligand (0.30 g, 0.50 mmol) in THF (15 mL). After 2 h, a cloudy suspension formed. The completion of the deprotonation reaction was verified by $^{31}P\{^1H\}$ NMR spectroscopy. The insoluble potassium salt was removed by centrifugation and $LuCl_3$ (75 mg, 0.27 mmol) was added. After 4 h of stirring at 298 K, potassium ethoxide (23 mg, 0.27 mmol) was added into the mixture, giving a cloudy suspension. Stirring was continued for 4 h, after which time the solid was removed by centrifugation. The solvent was evaporated *in vacuo* and the residue was crystallised or precipitated from a mixture of cyclohexane and hexane (4 mL), giving compound **2** as colourless crystals (220 mg, 0.20 mmol, 75 %).

^1H NMR (400 MHz, toluene-d_8, 298 K) δ (ppm): 7.72 (bm, 7H, C*H*(PPh_2)), 7.55 (d, 2H, C_bH, $^4J_{H,H}$=2.3 Hz), 6.95-7.14 (bm, hidden by toluene-d_8 signal, C*H*(PPh_2)), 6.90 (dd, 2H, C_dH, $^4J_{H,H}$=2.4 Hz, $^3J_{P,H}$=16.0 Hz), 4.55 (q, 2H, CH_2CH_3, $^3J_{H,H}$=6.8 Hz), 3.15 (m, 4H, P=N-CH_2-CH_2), 2.82 (m, 4H, P=N-CH_2-CH_2), 2.34 (bm, 1H, N*H*), 1.53 (t, 3H, CH_2CH_3, $^3J_{H,H}$=6.8 Hz), 1.48 (s, 18H, *t*Bu), 1.27 (s, 18H, *t*Bu); $^{31}P\{^1H\}$ NMR (161.9 MHz, toluene-d_8, 298 K) δ (ppm): 34.8 (s, P); $^{13}C\{^1H\}$ NMR (100 MHz, benzene-d_6, 298 K) δ (ppm): 169.7 (d, $^2J_{P,C}$=3.6 Hz, C^{IV}-O), 140.8 (d, $^3J_{P,C}$=8.0 Hz, $C_{c,a}^{IV}$), 134.1 (d, $^3J_{P,C}$=15.0 Hz, $C_{c,a}^{IV}$), 133.5 (d, $^{2/3}J_{P,C}$=9.9 Hz, *m*-or *o*-CH(PPh_2)) 133.4 (d, $^{2/3}J_{P,C}$=9.0 Hz, *m*-or *o*-CH(PPh_2)), 132.4 (d, $^1J_{P,C}$=75.0 Hz, C^{IV}-P), 131.4 (s, *p*-CH(PPh_2)), 131.2 (s, *p*-CH(PPh_2)), 128.5 (d, $^{2/3}J_{P,C}$=10.0 Hz, *m*-or *o*-CH(PPh_2)), 128.2 (s, C_bH), 128.0 (d, $^{2/3}J_{P,C}$=12.0 Hz, *m*-or *o*-CH(PPh_2)), 126.8 (d, $^1J_{P,C}$=13.0 Hz, C_dH), 110.0 (d, $^1J_{P,C}$=125.0 Hz, C^{IV}-P), 63.2 (s, O-CH_2CH_3), 53.2 (d, $^2J_{P,C}$=13.0 Hz, P^{IV}-N-CH_2), 48.5 (d, $^3J_{P,C}$=3.0 Hz, P^{IV}-N-CH_2-CH_2), 35.9 (s, $C^{IV}(CH_3)_3$), 34.2 (s, $C^{IV}(CH_3)_3$), 32.0 (s, $C^{IV}(CH_3)_3$), 30.1 (s, O-CH_2CH_3); Anal. Calc. ($C_{58}H_{74}N_3O_4P_2Lu$): C, 63.44; H, 6.79; N, 3.83. Found: C, 63.50; H, 6.62; N, 3.84.

Compound 3

Potassium *bis*(trimethylsilyl)amide (266 mg, 1.30 mmol) was added into a slurry of the phosphasalen pro-ligand (0.30 g, 0.50 mmol) in THF (15 mL). After 2 h, a cloudy suspension formed. The completion of the deprotonation reaction was verified by $^{31}P\{^1H\}$ NMR spectroscopy. The insoluble potassium salt was removed by centrifugation and $LaCl_3$ (66 mg, 0.27 mmol) was added. After 4 h of stirring at 298 K, potassium *tert*-butoxide (30 mg, 0.27 mmol) was added into the mixture, giving a cloudy suspension. Stirring was continued for 4 h, after which time the solid was removed by centrifugation. The solvent was evaporated *in vacuo* and the residue was crystallised or precipitated from a mixture of cyclohexane and hexane (4 mL), giving compound **3** as colourless crystals (100 mg, 0.10 mmol, 34 %).

^1H NMR (400 MHz, toluene-d_8, 360 K) δ (ppm): 7.46-7.74 (bm), 7.10-7.17 (bm), 6.97-7.15 (broad peaks under toluene-d_8 signals), 6.79 (dd, C_dH, $^4J_{H,H}$=2.4 Hz, $^3J_{P,H}$=15.2 Hz), 3.27 (bm, CH_2), 2.99 (bm, CH_2), 2.83 (bm, CH_2), 2.43 (bm, CH_2), 2.33 (bm, N*H*), 1.49 (bs, *t*Bu), 1.44 (bs, O*t*Bu), 1.23 (s, *t*Bu), 1.21 (s, *t*Bu), 1.20 (s, *t*Bu); $^{31}P\{^1H\}$ NMR (161.9 MHz, toluene-d_8, 298 K) δ (ppm): 34.8 (s, P); $^{13}C\{^1H\}$ NMR (100 MHz, THF-d_8, 298 K) δ (ppm): 170.0 (d, $^2J_{P,C}$=3.5 Hz, C^{IV}-O), 139.6 (d, $^3J_{P,C}$=8.0 Hz, $C_{c,a}^{IV}$), 133.9 (d, $^3J_{P,C}$=15.0 Hz, $C_{c,a}^{IV}$), 134.0 (d, $^{2/3}J_{P,C}$=9.9 Hz, *m*-or *o*-CH(PPh_2)) 133.8 (d, $^{2/3}J_{P,C}$=9.0 Hz, *m*-or *o*-CH(PPh_2)), 133.1 (d, $^1J_{P,C}$=39.0 Hz, C^{IV}-P), 132.0 (s, *p*-CH(PPh_2)), 131.9 (s, *p*-CH(PPh_2)), 129.5 (d, $^{2/3}J_{P,C}$=11.0 Hz, *m*-or *o*-CH(PPh_2)), 129.2 (d, $^{2/3}J_{P,C}$=11.0 Hz, *m*-or *o*-CH(PPh_2)), 128.7 (s, C_bH), 127.0 (d, $^1J_{P,C}$=12.6 Hz, C_dH), 114.0 (d, $^1J_{P,C}$=119.0 Hz, C^{IV}-P), 71.6 (s, O-$C^{IV}(CH_3)_3$), 54.1 (d, $^2J_{P,C}$=19.0 Hz, P^{IV}-N-CH_2), 48.8 (d, $^3J_{P,C}$=5.0 Hz, P^{IV}-N-CH_2-CH_2), 36.1 (s, $C^{IV}(CH_3)_3$), 35.2 (s, O-$C^{IV}(CH_3)_3$), 34.7 (s, $C^{IV}(CH_3)_3$), 32.2 (s, $C^{IV}(CH_3)_3$), 30.8 (s, $C^{IV}(CH_3)_3$); Anal. Calc. ($C_{60}H_{78}N_3O_4P_2La$): C, 66.11; H, 7.21; N, 3.85. Found: C, 65.92; H, 7.35; N, 3.94.

General Polymerization Procedure

In a glove box, a tube was loaded with *rac*-lactide (288 mg, 2 mmol) and dissolved in THF (1.8 mL). A stock solution of initiator (0.2 mL, 0.02 M) was injected into the reaction, such that the overall

concentration of lactide was 1 M and of initiator was 2 mM. Aliquots were taken from the reaction under a nitrogen atmosphere, quenched with wet hexane (1-2 mL) and the solvent was allowed to evaporate. The crude product was analysed by ^{1}H NMR and homonuclear decoupled ^{1}H NMR spectroscopy and GPC-MALLS. The conversion of LA to PLA was determined by integration of the methyne proton peaks of the ^{1}H NMR spectra, δ 5.00 – 5.30. The P_s or P_i value was determined by integration of the methyne region of the homonuclear decoupled ^{1}H NMR spectrum, δ 5.1 – 5.24. The methyne proton region was deconvoluted using MestReNova software. The PLA number-averaged molecular weight, M_n, and polydispersity index (M_w/M_n; PDI) were determined using Gel Permeation Chromatography (GPC). Calculated M_n values, where exogenous alcohol was added, were determined on the basis of one chain growing per initiator; *tert*-butyl alcohol/alkoxide is a poor initiator and inefficient chain-transfer agent.

General polymerization Procedure at Low Temperature

In a glove box, a tube was loaded with *rac*-lactide (115 mg, 0.80 mmol) and dissolved in cold THF (1.4 mL). A stock solution of initiator (0.2 mL, 0.02 M) was injected into the reaction, such that the overall concentration of lactide was 0.5 M. The tube was then removed from the glovebox and placed on a stirrer plate in a freezer, such that the temperature was maintained at 257 K. After an allotted period of time the reaction was quenched with hexane (5 mL) and the solvent was allowed to evaporate. The resultant polymer was analysed as described above.

X-ray Crystallography

Crystal data for **1**: $C_{60}H_{78}LuN_3O_3P_2{\cdot}3(C_6H_{12})$, *M* = 1378.63, triclinic, *P*-1 (no. 2), *a* = 10.7519(2), *b* = 15.3848(3), *c* = 23.6010(5) Å, α = 80.1202(19), β = 84.4658(19), γ = 76.7280(18)°, *V* = 3736.72(15) $Å^3$, *Z* = 2, ρ_{calcd} = 1.225 g cm^{-3}, $\mu(Cu_{K\alpha})$ = 3.280 mm^{-1}, *T* = 173 K, colourless platy needles, Agilent Xcalibur PX Ultra diffractometer; 14526 independent measured reflections (R_{int} = 0.0309), F^2 refinement,[5] R_1(obs) = 0.0554, wR_2(all) = 0.1506, 13434 independent observed absorption-corrected reflections [$|F_o| > 4\sigma(|F_o|)$, $2\theta_{max}$ = 148°], 849 parameters. CCDC 990905.

Crystal data for **2**: $C_{58}H_{74}LuN_3O_3P_2{\cdot}0.75(C_6H_{12}){\cdot}0.5(C_6H_{14})$, *M* = 1204.31, monoclinic, *I*2/a (no. 15), *a* = 22.3632(5), *b* = 20.5167(5), *c* = 30.5029(7) Å, β = 105.916(3)°, *V* = 13458.8(6) $Å^3$, *Z* = 8, ρ_{calcd} = 1.189 g cm^{-3}, $\mu(Cu_{K\alpha})$ = 3.575 mm^{-1}, *T* = 173 K, colourless needles, Agilent Xcalibur PX Ultra diffractometer; 19024 independent measured reflections, F^2 refinement,[5] R_1(obs) = 0.1015, wR_2(all) = 0.2705, 14526 independent observed absorption-corrected reflections [$|F_o| > 4\sigma(|F_o|)$, $2\theta_{max}$ = 147°], 773 parameters. CCDC 990906.

Crystal data for **3**: $C_{60}H_{78}LaN_3O_3P_2{\cdot}0.5(C_6H_{14}){\cdot}0.5(C_6H_{12})$, *M* = 1175.26, monoclinic, $P2_1/c$ (no. 14), *a* = 28.1717(5), *b* = 15.3578(3), *c* = 29.8704(8) Å, β = 94.948(2)°, *V* = 12875.4(5) $Å^3$, *Z* = 8 (two independent molecules), ρ_{calcd} = 1.213 g cm^{-3}, $\mu(Cu_{K\alpha})$ = 5.933 mm^{-1}, *T* = 173 K, colourless tablets, Agilent Xcalibur PX Ultra diffractometer; 24639 independent measured reflections (R_{int} = 0.0389), F^2 refinement,[5] R_1(obs) = 0.0562, wR_2(all) = 0.1847, 19468 independent observed absorption-corrected reflections [$|F_o| > 4\sigma(|F_o|)$, $2\theta_{max}$ = 148°], 1487 parameters. CCDC 990907.

The X-ray crystal structure of 1

The N(11)–H hydrogen atom in the structure of **1** was found from a ΔF map and refined freely subject to an N–H distance constraint of 0.90 Å. The C(26)-based *tert*-butyl group was found to be disordered, and two orientations were identified of *ca*. 77 and 23 % occupancy. Their geometries were optimised, the thermal parameters of adjacent atoms were restrained to be similar, and only the non-hydrogen atoms of the major occupancy orientation were refined anisotropically (those of the minor occupancy orientation were refined isotropically). The C(36)-based phenyl ring showed signs of disorder, but a model using two partial occupancy orientations did not prove satisfactory, so instead a model with one geometrically idealised orientation was employed. The C(91)- and C(111)-based cyclohexane solvent molecules were both found to be disordered, the former in a general position and the latter across a centre of symmetry. In each case two unique orientations were identified, of *ca*. 74 and 26 % occupancy for the C(91)-based molecule, and of *ca*. 25 % occupancy each for the C(111)-based molecule. For this latter case, two further orientations of *ca*. 25 % occupancy are generated by operation of the centre of symmetry. The geometries of all four unique orientations were optimised, the thermal parameters of adjacent atoms were restrained to be similar, and only the non-hydrogen atoms of the major occupancy orientation of the C(91)-based molecule were refined anisotropically (the remainder were refined isotropically).

The X-ray crystal structure of 2

The crystal of **2** that was studied was found to be a two component twin in a *ca*. 57:43 ratio, with the two lattices related by the approximate twin law [1.00 0.00 0.00 0.00 1.03 0.00 0.00 0.00 1.00]. The N(11)–H hydrogen atom could not be located from ΔF maps, and so was added in an idealised position using an N–H distance constraint of 0.90 Å. The C(26)- and C(54)-based *tert*-butyl groups were found to be disordered. In each case two orientations were identified, of *ca*. 73:27 and 72:28 occupancy respectively. The geometries of all four orientations were optimised, the thermal parameters of adjacent atoms were restrained to be similar, and only the non-hydrogen atoms of the major occupancy orientations were refined anisotropically (the remainder were refined isotropically). The C(71)-based ethyl group was found to be disordered. Two orientations were identified, of *ca*. 63 and 37 % occupancy respectively, their geometries were optimised, the thermal parameters of adjacent atoms were restrained to be similar, and only the non-hydrogen atoms of the major occupancy orientation were refined anisotropically (the remainder were refined isotropically). The

C(81)-based *n*-hexane solvent molecule was found to be disordered across a C_2 axis. This disorder was modelled by using one complete 50 % occupancy orientation, allowing the C_2 axis to generate a second orientation. The geometry of the unique orientation was optimised, and the non-hydrogen atoms were refined anisotropically. The C(91)-based cyclohexane solvent molecule was found to be disordered across a centre of symmetry. Two unique orientations were identified of *ca*. 32 and 18 % occupancy, with the operation of the centre of symmetry generating two further orientations of the same occupancies. The geometries of the two unique orientations were optimised, the thermal parameters of adjacent atoms were restrained to be similar, and all of the atoms were refined isotropically. The C(101)-based cyclohexane solvent molecule was found to be disordered across a C_2 axis. This disorder was modelled by using one complete orientation, allowing the C_2 axis to generate a second orientation. Based on the thermal parameters, the occupancy of the unique orientation was set at 25 %. The geometry of the unique orientation was optimised, and the non-hydrogen atoms were refined anisotropically.

The X-ray crystal structure of 3

The structure of **3** was found to contain two crystallographically independent molecules (**A** and **B**) in the asymmetric unit. The N(11)–H hydrogen atom in each molecule could not be located from ΔF maps, and so they were added in idealised positions using an N–H distance constraint of 0.90 Å. In molecule **A** the CH_2–CH_2 linkage between N(8) and N(11) was found to be disordered. Two orientations were identified of *ca*. 73 and 27 % occupancy, their geometries were optimised, the thermal parameters of adjacent atoms were restrained to be similar, and only the non-hydrogen atoms of the major occupancy orientation were refined anisotropically (those of the minor occupancy orientation were refined isotropically). The C(26A)-, C(71A)-, C(26B)-, C(54B)- and C(71B)-based *tert*-butyl groups were all found to be disordered, and in each case two orientations were identified, of *ca*. 82:18, 67:33, 61:39, 82:18 and 79:21 occupancy respectively. The geometries of all ten orientations were optimised, the thermal parameters of adjacent atoms were restrained to be similar, and only the non-hydrogen atoms of the major occupancy orientation were refined anisotropically (those of the minor occupancy orientation were refined isotropically). The C(81)-based *n*-hexane, and the C(91)-based cyclohexane, solvent molecules were both found to be disordered. In each case three orientations were identified, of *ca*. 43:36:21 and 47:28:25 occupancy respectively. In both instances the geometries of the three orientations were optimised, the thermal parameters of adjacent atoms were restrained to be similar, and all of the atoms were refined isotropically.

Table S1: Comparative selected bond lengths (Å) and angles (°) for **1**, **2** and **3** (**A** and **B**).

	**1** **[M = Lu, R_1 = *t*-Bu]**	**2** **[M = Lu, R_1 = Et]**	**3** **[M = La, R_1 = *t*-Bu]**	
			**mol A**	**mol B**
M–O(1)	2.182(3)	2.157(6)	2.350(3)	2.336(4)
M–N(8)	2.360(3)	2.392(8)	2.574(4)	2.551(5)
M–N(11)	2.486(4)	2.515(8)	2.705(4)	2.732(5)
M–N(14)	2.395(3)	2.365(8)	2.639(4)	2.602(4)
M–O(21)	2.142(3)	2.143(6)	2.327(4)	2.332(4)
M–O(70)	2.060(3)	2.079(7)	2.196(4)	2.191(4)
O(1)···O(21)	3.074(4)	3.107(9)	3.603(5)	3.354(5)
O(1)–M–N(8)	77.44(11)	76.6(2)	72.11(13)	72.94(14)
O(1)–M–N(11)	118.56(11)	117.6(3)	121.06(13)	126.18(14)
O(1)–M–N(14)	158.87(12)	159.3(3)	149.44(13)	146.96(13)
O(1)–M–O(21)	90.60(11)	92.5(3)	100.77(13)	91.87(13)
O(1)–M–O(70)	99.36(13)	98.0(3)	99.78(14)	104.56(16)
N(8)–M–N(11)	68.99(12)	68.8(3)	64.14(13)	63.72(15)
N(8)–M–N(14)	88.89(12)	90.3(3)	86.99(13)	90.23(14)
N(8)–M–O(21)	109.04(13)	115.5(3)	125.66(13)	121.64(14)
N(8)–M–O(70)	144.52(14)	141.9(3)	127.11(16)	123.71(18)
N(11)–M–N(14)	69.68(12)	70.7(3)	64.69(13)	64.97(14)
N(11)–M–O(21)	148.19(11)	149.1(3)	136.45(12)	137.49(14)
N(11)–M–O(70)	82.61(13)	81.4(3)	78.89(15)	76.94(17)
N(14)–M–O(21)	78.60(11)	78.6(3)	73.21(13)	72.69(12)
N(14)–M–O(70)	101.08(13)	102.1(3)	110.67(15)	108.41(16)
O(21)–M–O(70)	106.30(13)	102.3(3)	107.22(15)	114.62(17)

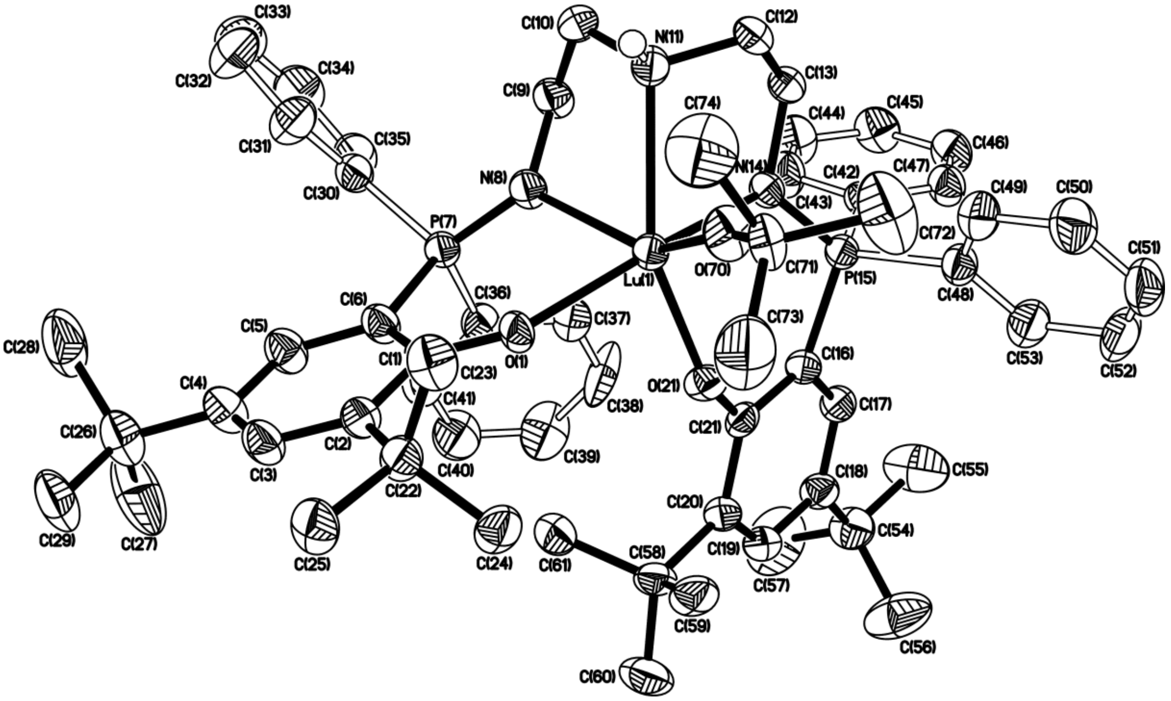

Figure S1: The crystal structure of **1** (50% probability ellipsoids).

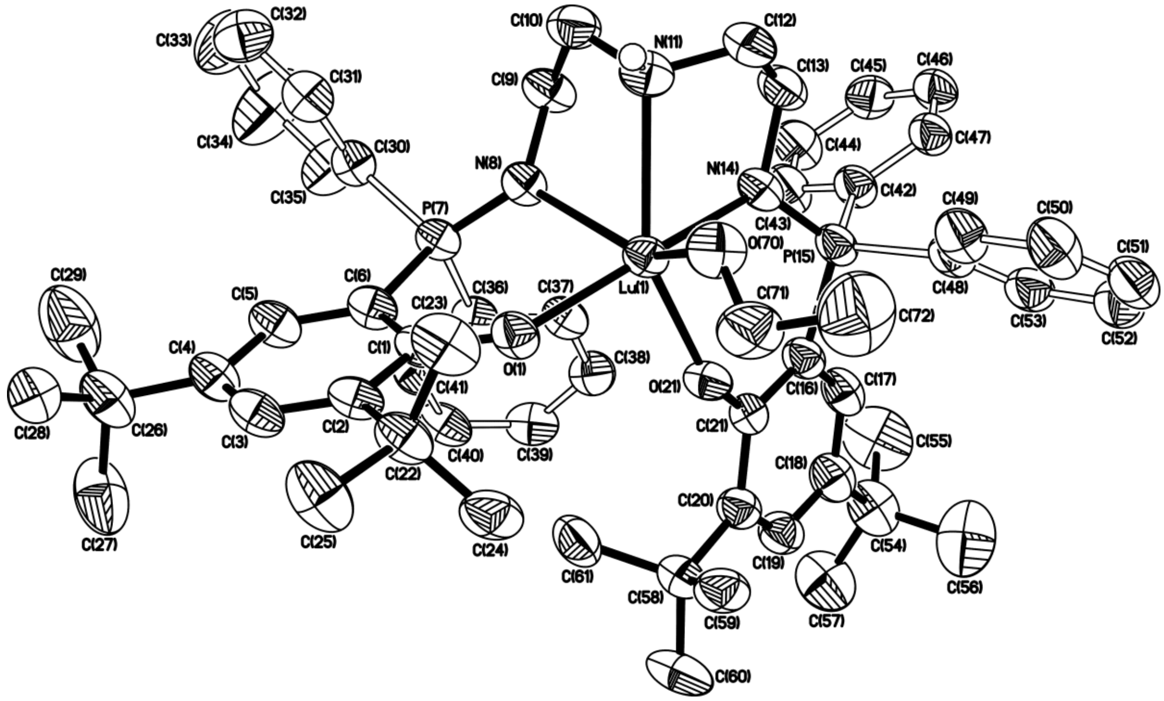

Figure S2: The crystal structure of **2** (50% probability ellipsoids).

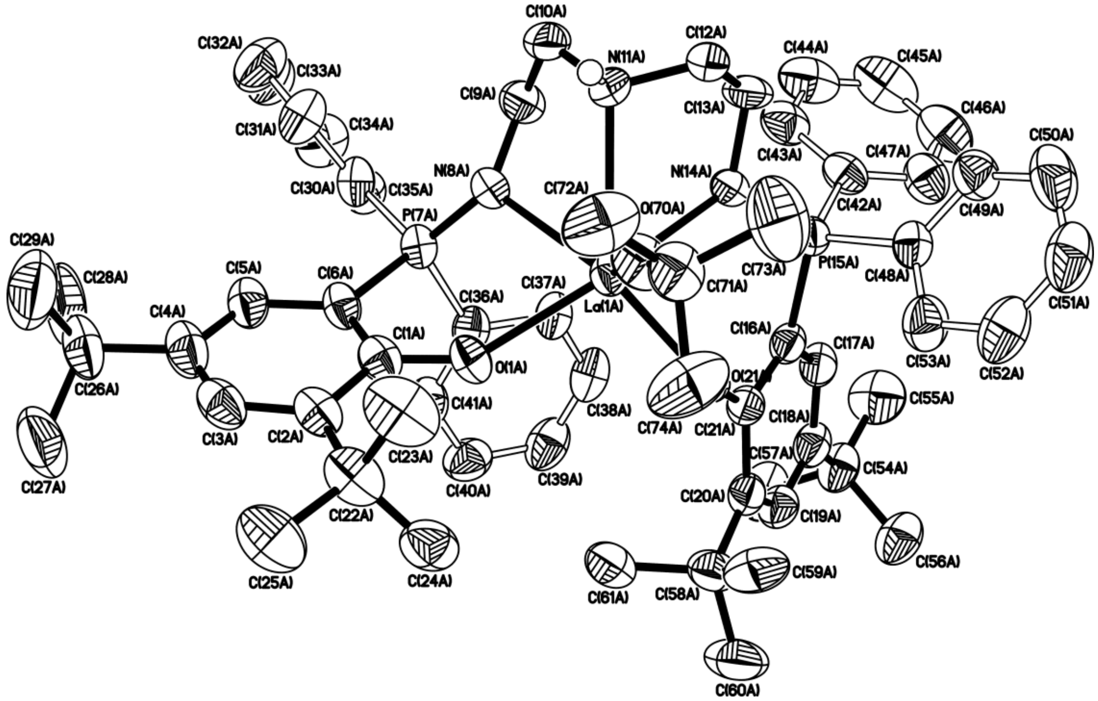

Figure S3: The crystal structure of one (**3-A**) of the two independent molecules present in the crystal of **3** (50% probability ellipsoids).

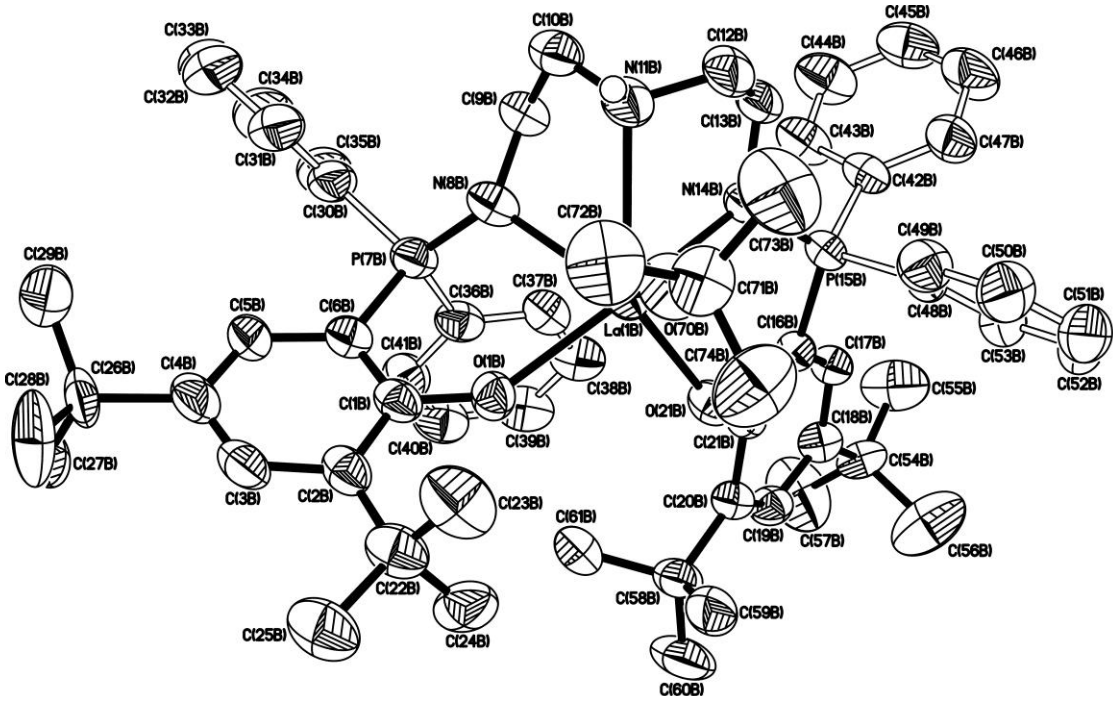

Figure S4: The crystal structure of one (**3-B**) of the two independent molecules present in the crystal of **3** (50% probability ellipsoids).

NMR Spectra of Compounds 1-3

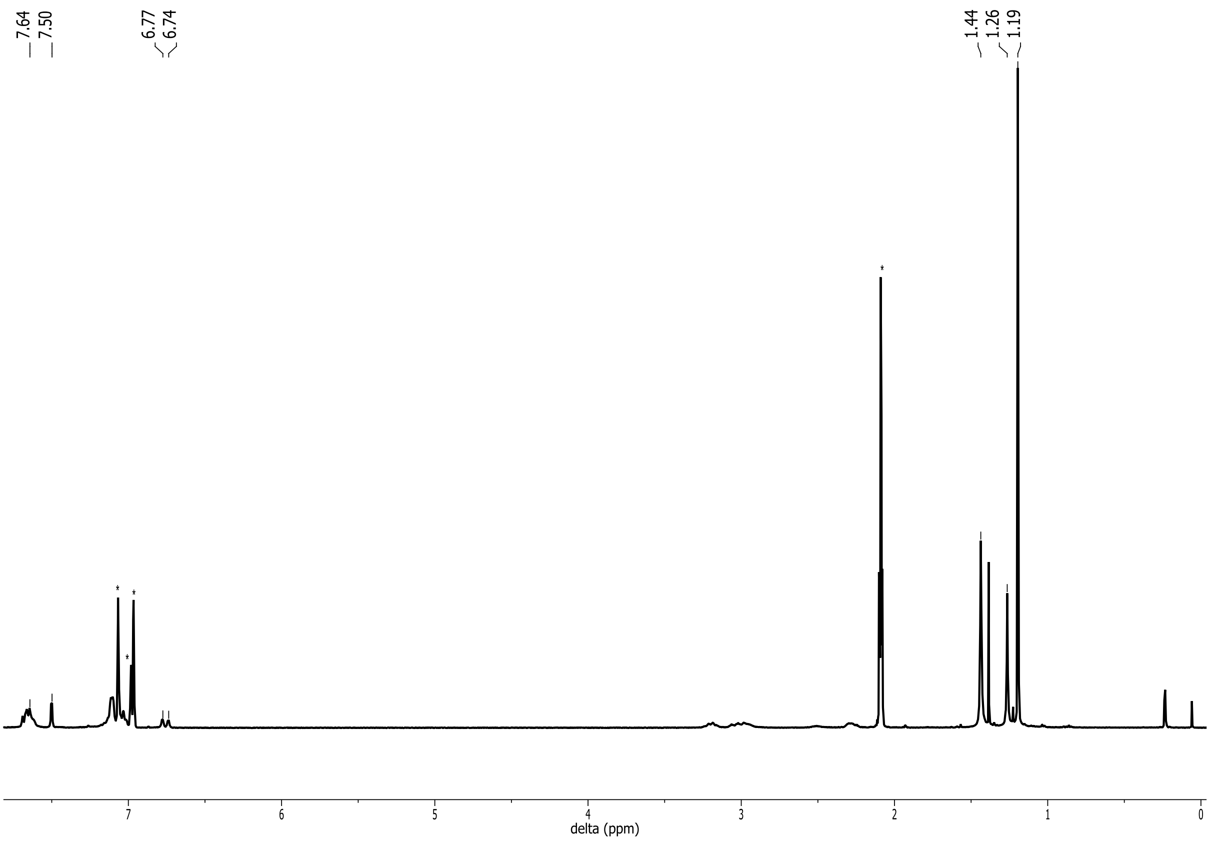

Figure S5: ^{1}H NMR spectrum of compound **1** at 360 K in toluene-d_8; residual solvent peaks marked with asterisks.

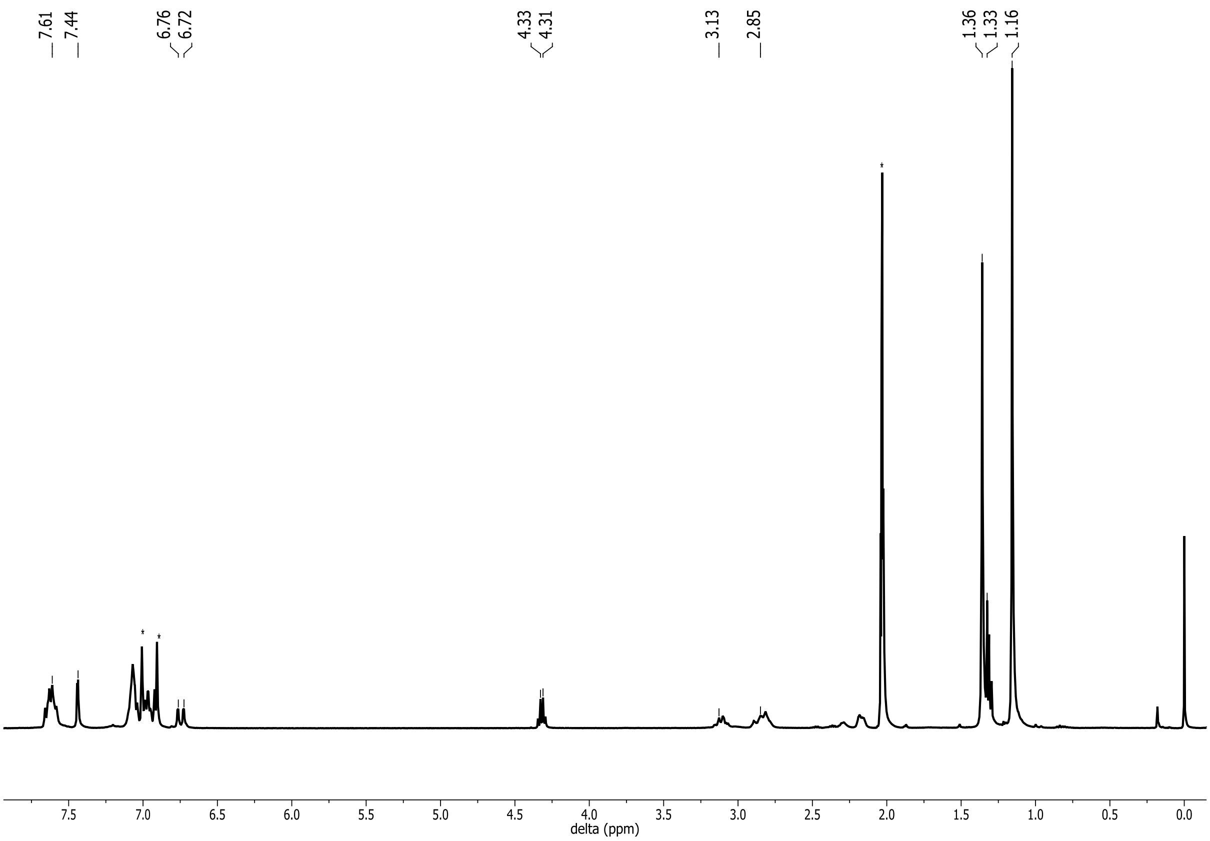

Figure S6: ^{1}H NMR spectrum of compound **2** at 360 K in toluene-d_8; residual solvent peaks marked with asterisks.

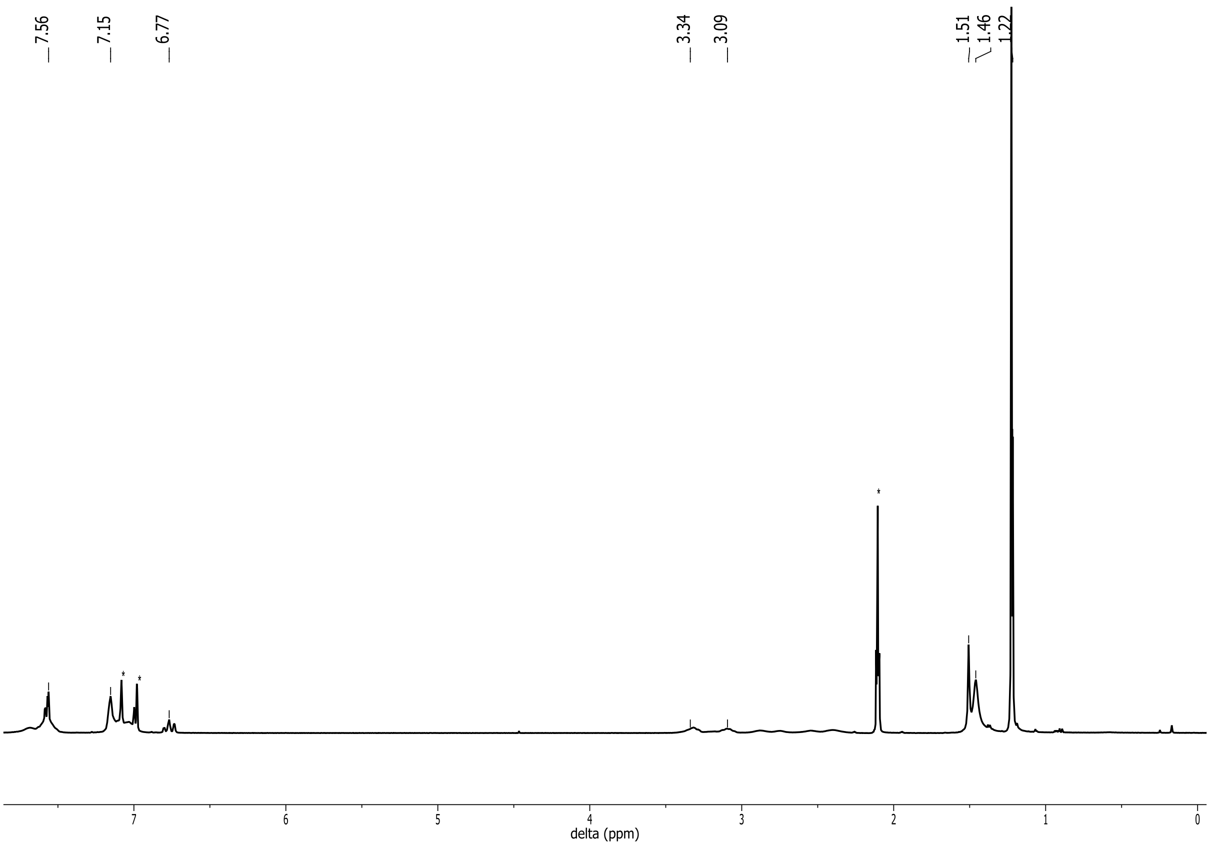

Figure S7: ^{1}H NMR spectrum of compound **3** at 360 K in toluene-d_8; residual solvent peaks marked with asterisks.

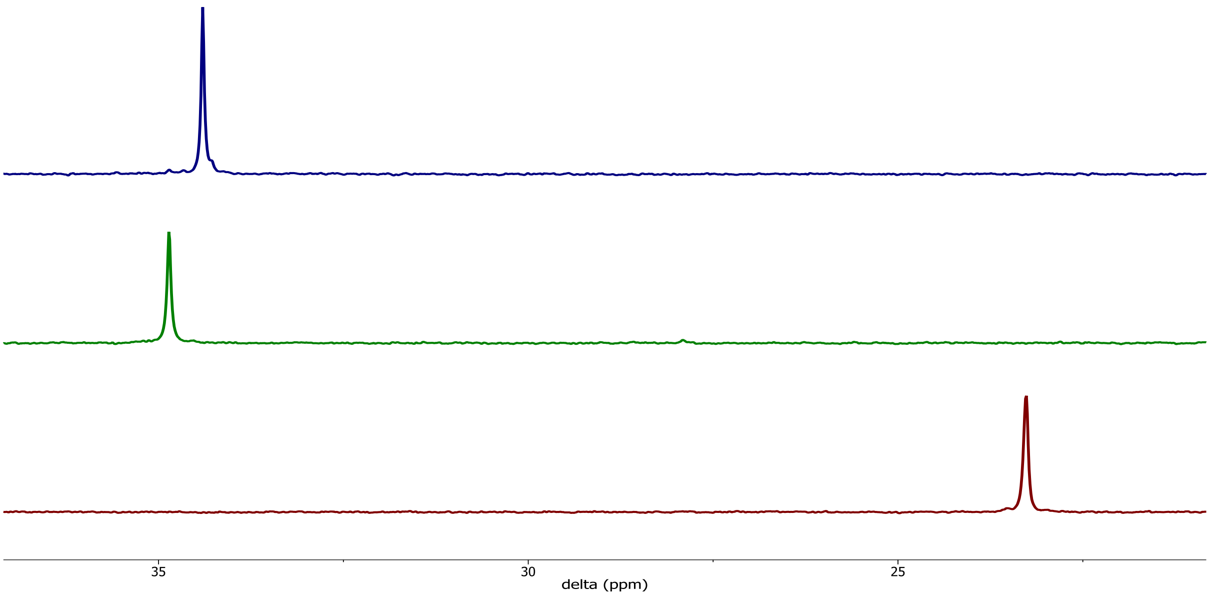

Figure S8: A stack plot of the $^{31}P\{^{1}H\}$ NMR spectra showing the formation of compound **2**; after addition of KHMDS (bottom), after addition of $LuCl_3$ (middle), after addition of KOEt (top).

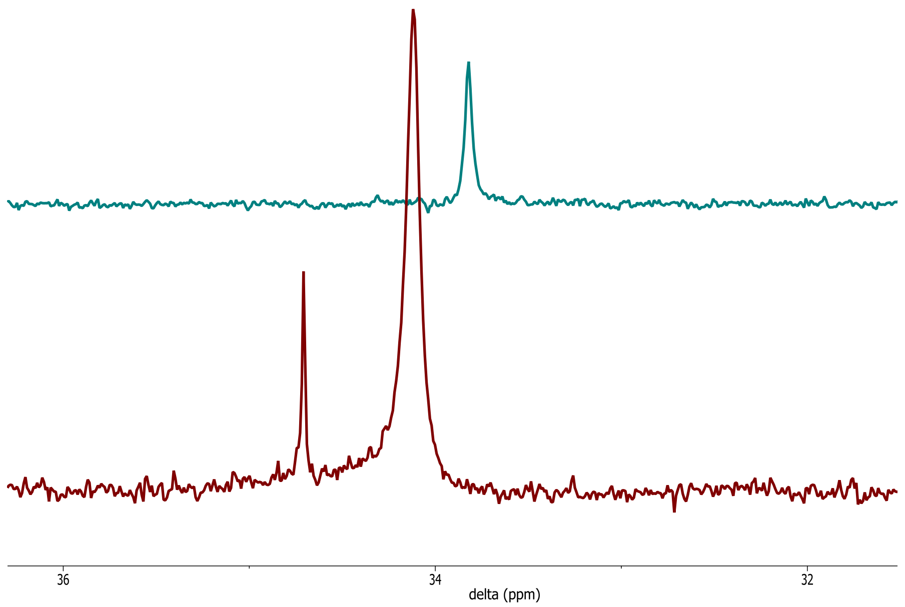

Figure S9: The $^{31}P\{^{1}H\}$ NMR spectra of compound **1** at 298 K (bottom) and at 360 K (top).

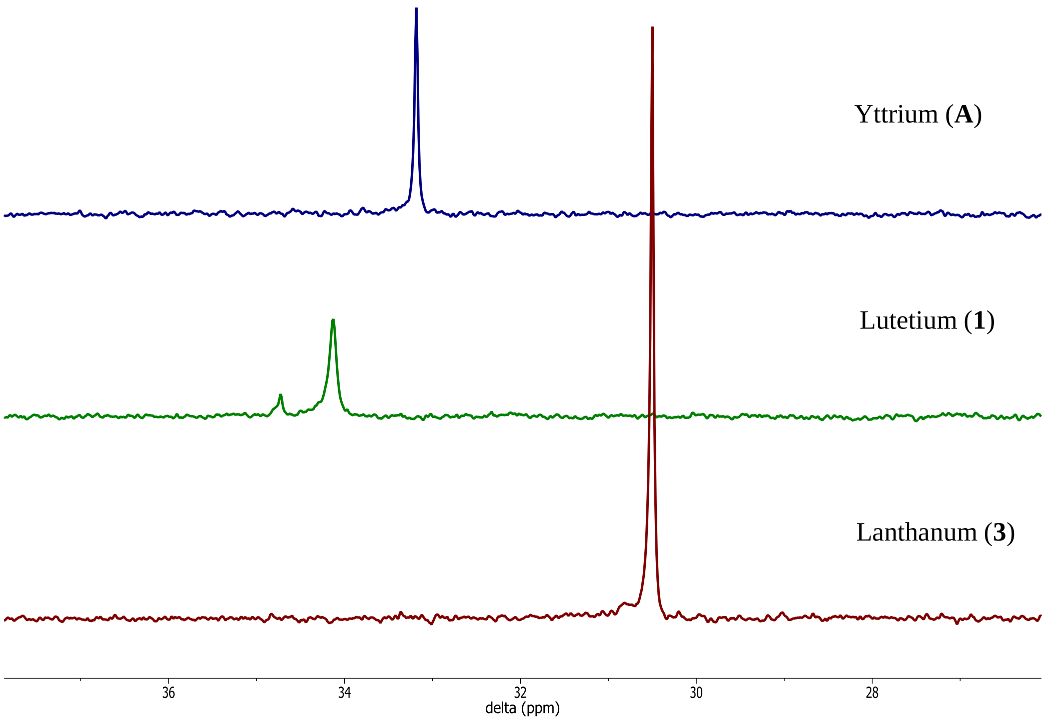

Figure S10: A comparison of the signals observed in the $^{13}P\{^{1}H\}$ NMR spectra (at 298 K, in toluene-d_8) of compounds **A**, **1** and **3**.

Polymerization Data using Initiators 1-3

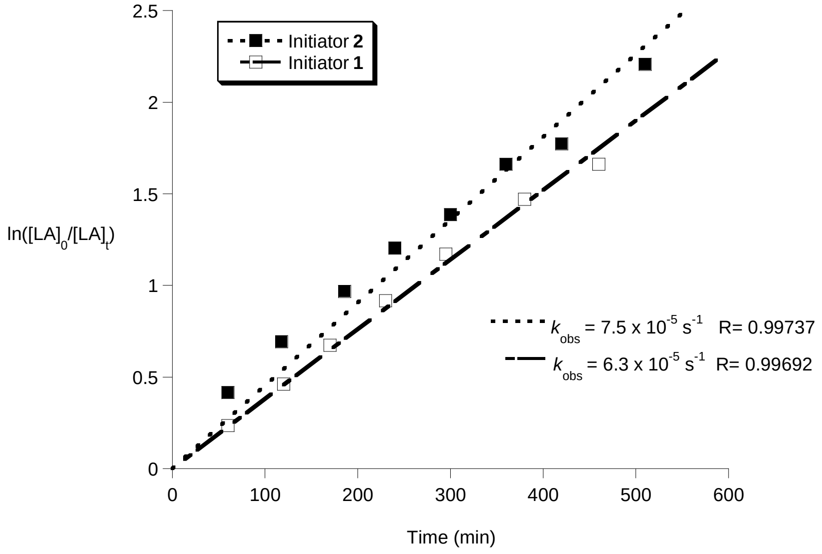

Figure S11: Plot of ln($[LA]_0$/$[LA]_t$) vs. time for initiators **1** and **2**. Conditions: $[LA]_0$ = 1 M, 1:1:100 [**1**]:[*i*PrOH]:[LA] and 1:100 [**2**]:[LA], THF, 298 K.

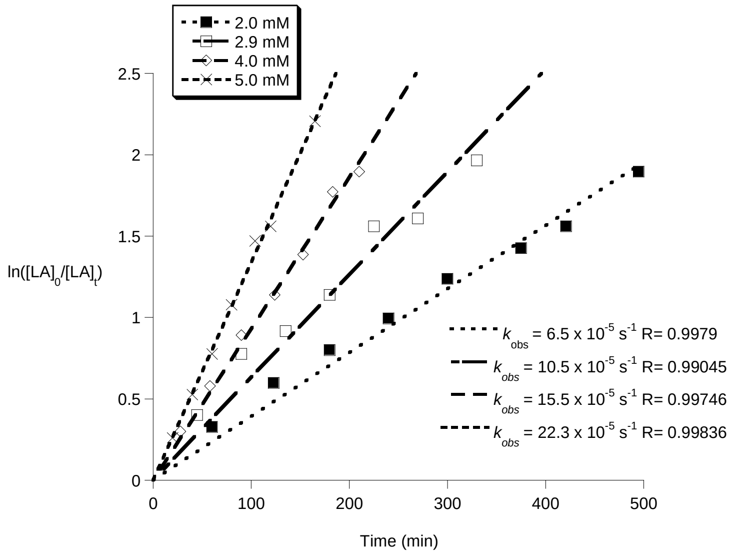

Figure S12: Plots of ln($[LA]_0$/$[LA]_t$) vs. time using initiator **2** at different catalyst concentrations; 2 mM (black squares), 2.9 mM (white squares), 4 mM (white diamonds), 5 mM (crosses). Conditions: $[LA]_0$ = 1 M, THF, 298 K.

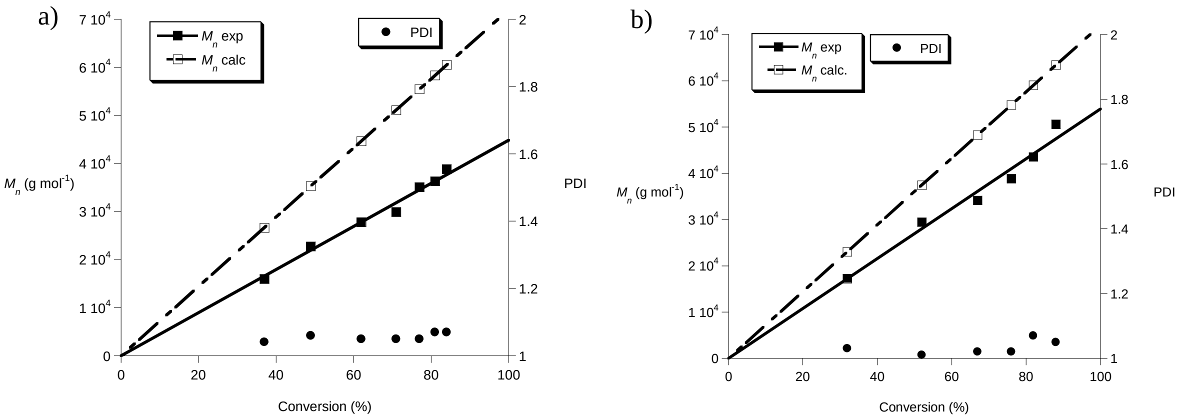

Figure S13: Plots of M_n versus % conversion a) Using initiator **1** with 1 equiv. *i*PrOH b) Using initiator **2**.

Homonuclear decoupled ^{1}H {^{1}H} NMR spectra

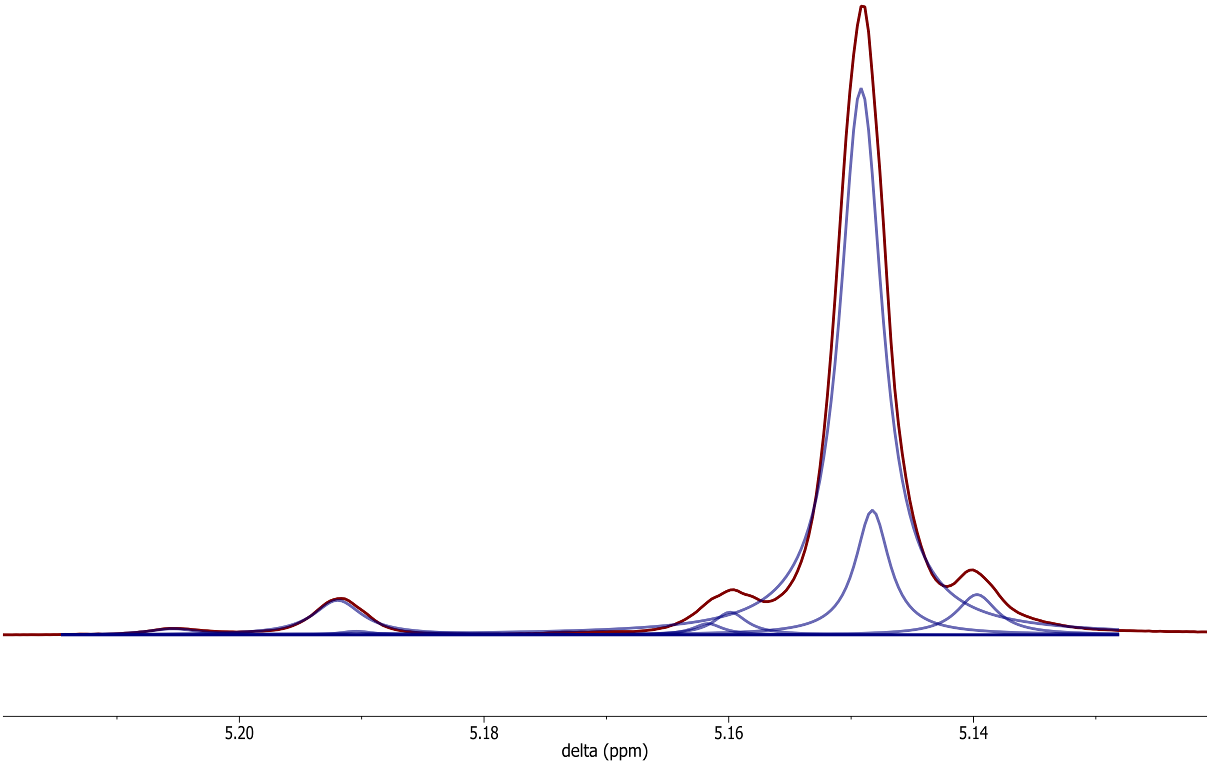

Figure S14: The methyne region of the $^{1}H\{^{1}H\}$ NMR spectrum of PLA in $CDCl_3$. Reagents and conditions: 1:200 [**2**]:[LA], 0.5 M [LA], 257 K (P_i = 0.89).

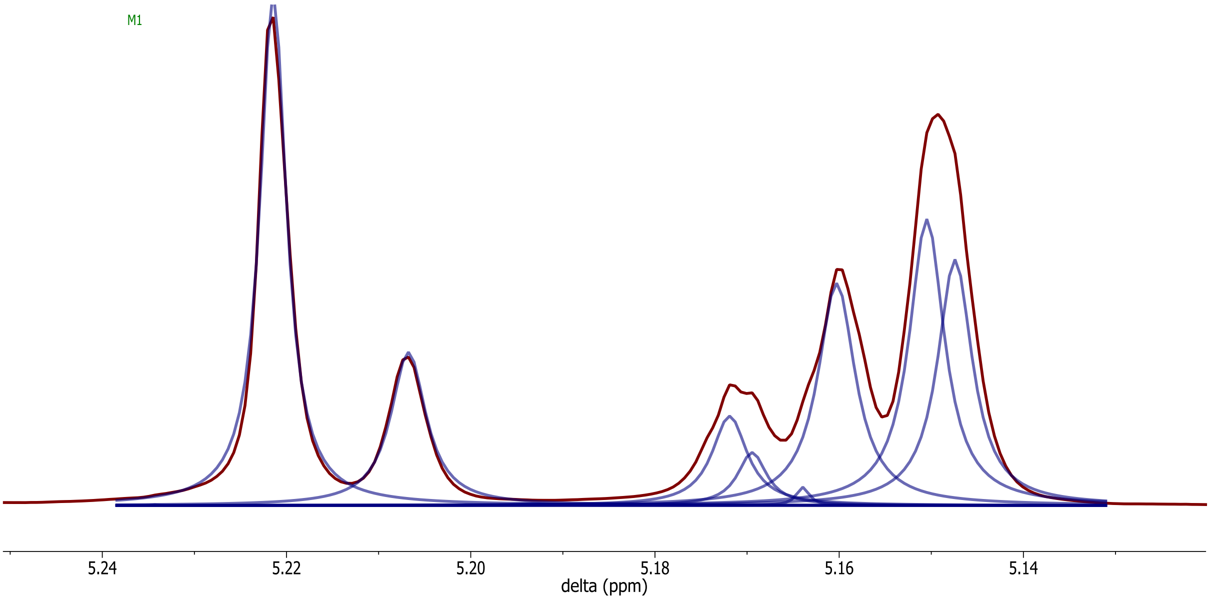

Figure S15: The methyne region of the $^{1}H\{^{1}H\}$ NMR spectrum of PLA in $CDCl_3$. Reagents and conditions: 1:2:1000 [**3**]:[*i*PrOH]:[LA], 1 M [LA], 298 K (P_s = 0.72).

DSC Data

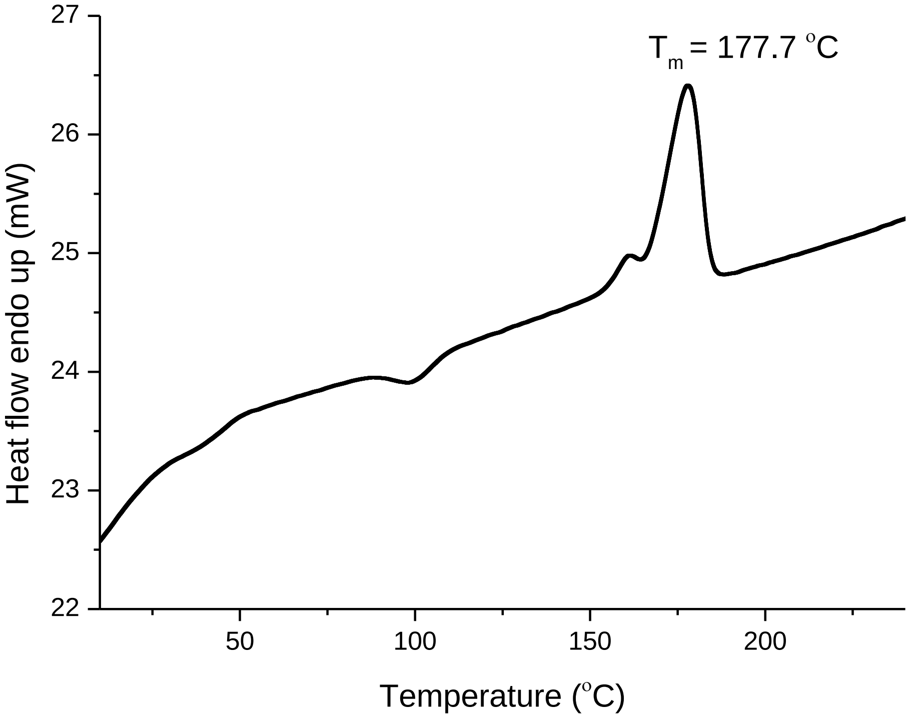

Figure S16: DSC trace of PLA, P_i = 0.89, M_n = 46,300 gmol^{-1}. Second heating curve shown, cooling curves omitted for clarity.

ROESY NMR Spectra

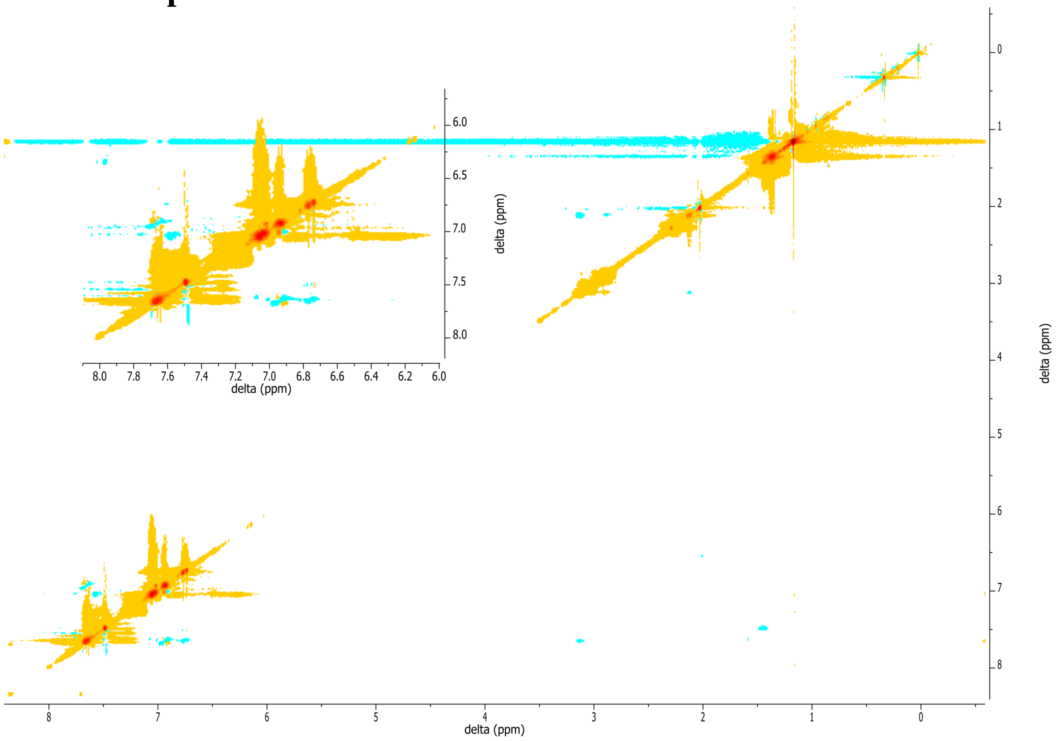

Figure S17: ROESY NMR spectrum of compound **2** at 298 K in toluene-d_8.

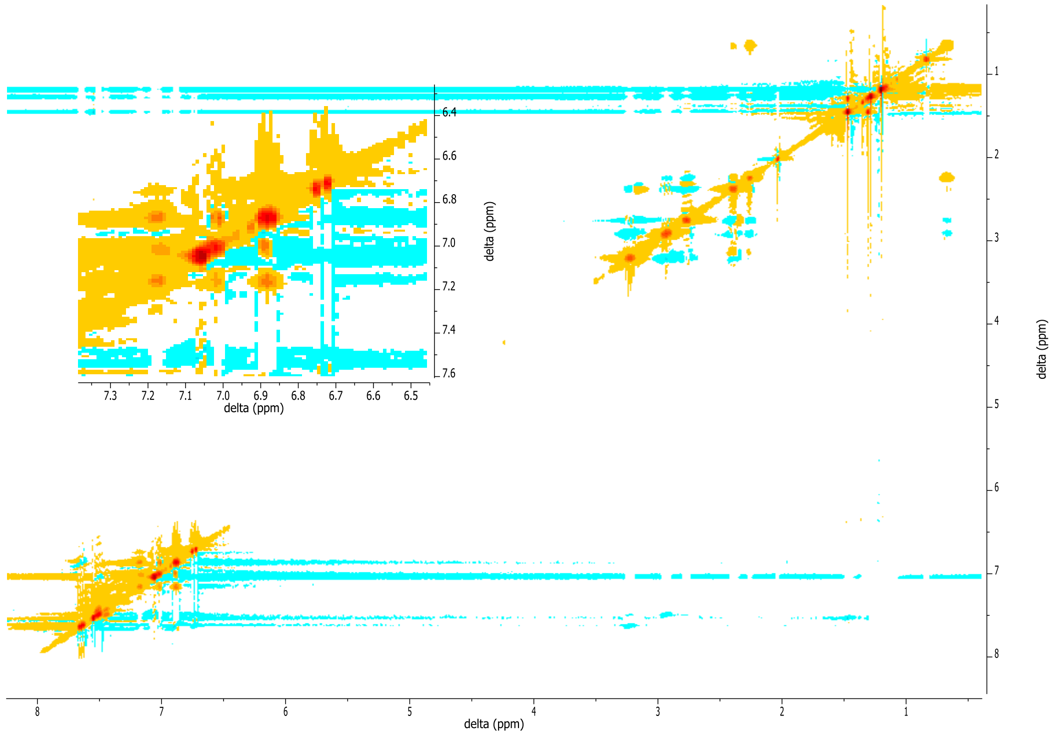

Figure S18: ROESY NMR spectrum of compound **3** at 298 K in toluene-d_8.

Table S2: Expanded table of results of polymerization data obtained using initiators **1-3**

I	Loading	Conc.	*i*PrOH	Solvent	T (K)	Time (h)	Conversion (%)[a]	k_{obs} (10^{-5} s^{-1})[b]	$M_{n, exp}$ (g/mol)[c]	$M_{n\ calc}$ (g/mol)	PDI[c]	P_i[d]
**1**	1/500	1		THF	298	8	81	6.3	101,700	58,300	1.06	0.80
**1**	1/500	1	1	THF	298	9	84	6.3	38,900	60,500	1.07	0.75
**1**	1/500	0.75	1	THF	257	48	67		38,600	48,200	1.04	0.85
**1**	1/500	0.75	0.5	THF	257	72	84		69,600	60,500	1.02	0.84
**1**	1/200	0.5	0.5	THF	257	48	90		36,000	25,900	1.02	0.83
**2**	1/500	1		THF	298	8.25	86	6.5	53,400	61,900	1.02	0.82
**2**	1/350	1		THF	298	5.5	86	10.2	38,300	43,300	1.09	0.82
**2**	1/250	1		THF	298	3.5	86	15.6	34,900	31,000	1.02	0.84
**2**	1/200	1		THF	298	2.75	89	22.3	27,800	25,600	1.05	0.81
**2**	1/500	0.75		THF	257	72	75		46,300	54,000	1.01	0.89
**2**	1/200	0.5		THF	257	48	81		22,800	23,300	1.02	0.89
**3**	1/500	0.75	1	THF	298	20 secs	98		57,300	70,600	1.05	0.28
**3**	1/1000	1	2	THF	298	20 secs	93		58,000	133,900	1.03	0.28

[a] determined by integration of the methine region of the ^{1}H NMR spectrum (LA, 4.98 – 5.08 ppm; PLA, 5.09 – 5.24 ppm). [b] determined from the gradients of the plots of $\ln\{[LA]_0/[LA]_t\}$ versus time, where the average errors are 1−8%. [c] determined by GPC in THF vs. PS standards (M_n values are corrected with a 0.58 factor). [d] determined by analysis of all the tetrad signals in the methine region of the proton decoupled NMR spectrum.[4]

Table S3: Polymerization k_{obs} values at varying concentrations of initiator **2**; THF, 298 K, 1M [LA]

Loading	Concentration	k_{obs} (x 10^{-5} s^{-1})	
		Run 1	Run 2
1/200	5	22	22.3
1/250	4	15.6	16.8
1/350	2.86	10.5	10.16
1/500	2	7.5	6.5

Table S4: P_i values using compound **2** at different loadings; 298 K, THF, 1 M [LA]

**Loading**	**P_i**
1/200	0.81
1/250	0.84
1/350	0.82
1/500	0.82

References

[1] C. Bakewell, T.-P.-A. Cao, N. Long, X. F. Le Goff, A. Auffrant, C. K. Williams, *J. Am. Chem. Soc* **2012**, *134*, 20577-20580.

[2] A. Bax, D. G. Davis, *J.Magn. Reson.* **1985**, *63*, 207-213.

[3] A. Kowalski, A. Duda, S. Penczek, *Macromolecules* **1998**, *31*, 2114.

[4] J. Coudane, C. Ustariz-Peyret, G. Schwach, M. Vert, *J. Polym. Sci. Part A: Polym. Chem.* **1997**, *35*, 1651-1658.

[5] B. A. SHELXTL, Madison, WI; SHELX-97, G.M. Sheldrick, Acta Cryst., 2008, A64, 112-122; SHELX-2013, http://shelx.uni-ac.gwdg.de/SHELX/index.php.
